# Profile of Matrix-Remodeling Proteinases in Osteoarthritis: Impact of Fibronectin

**DOI:** 10.3390/cells9010040

**Published:** 2019-12-22

**Authors:** Selene Pérez-García, Mar Carrión, Irene Gutiérrez-Cañas, Raúl Villanueva-Romero, David Castro, Carmen Martínez, Isidoro González-Álvaro, Francisco J. Blanco, Yasmina Juarranz, Rosa P. Gomariz

**Affiliations:** 1 Departamento de Biología Celular, Facultad de Biología y Facultad de Medicina, Universidad Complutense de Madrid, 28040 Madrid, Spainmacarrio@ucm.es (M.C.); irgutier@ucm.es (I.G.-C.); ravillan@ucm.es (R.V.-R.); dcastr01@ucm.es (D.C.); cmmora@ucm.es (C.M.); yashina@ucm.es (Y.J.); 2 Servicio de Reumatología, Instituto de Investigación Médica, Hospital Universitario La Princesa, 28006 Madrid, Spain; isidoro.ga@ser.es; 3 Servicio de Reumatología, Instituto de Investigación Biomédica de A Coruña (INIBIC), Complexo Hospitalario Universitario de A Coruña, Sergas, Universidade da Coruña (UDC), 15006 A Coruña, Spain; fblagar@sergas.es

**Keywords:** osteoarthritis, fibronectin, proteinases, uPA, MMP, ADAMTS

## Abstract

The extracellular matrix (ECM) is a complex and specialized three-dimensional macromolecular network, present in nearly all tissues, that also interacts with cell surface receptors on joint resident cells. Changes in the composition and physical properties of the ECM lead to the development of many diseases, including osteoarthritis (OA). OA is a chronic degenerative rheumatic disease characterized by a progressive loss of synovial joint function as a consequence of the degradation of articular cartilage, also associated with alterations in the synovial membrane and subchondral bone. During OA, ECM-degrading enzymes, including urokinase-type plasminogen activator (uPA), matrix metalloproteinases (MMPs), and a disintegrin and metalloproteinase with thrombospondin motifs (ADAMTSs), cleave ECM components, such as fibronectin (Fn), generating fibronectin fragments (Fn-fs) with catabolic properties. In turn, Fn-fs promote activation of these proteinases, establishing a degradative and inflammatory feedback loop. Thus, the aim of this review is to update the contribution of ECM-degrading proteinases to the physiopathology of OA as well as their modulation by Fn-fs.

## 1. Introduction

Osteoarthritis (OA) is the most prevalent arthritic disease affecting the joints. While mainly related to aging, it is also associated with a diversity of risk factors including genetic predisposition, epigenetic factors, gender, obesity, exercise, work-related injury, and trauma. Irreversible and gradual loss of the articular cartilage remains the fundamental feature of OA pathophysiology [1,2,3,4]. However, in the course of the disease, all the joint tissues including synovium and bone undergo physical, functional, and metabolic alterations that comprise different cellular types as well as components of the extracellular cellular matrix (ECM). The ECM is a complex and specialized three-dimensional macromolecular network, present in nearly all tissues, which also interacts with cell surface receptors on joint resident cells. It is secreted, assembled, and modeled by the surrounding cells, providing physical support and organization to tissues. ECM is involved in many cell functions, providing cells with chemical and mechanical signals to regulate cell proliferation, survival, migration, and differentiation. However, changes in the composition and physical properties of the ECM lead to the development of many diseases, including cancer and rheumatic diseases, such as OA and rheumatoid arthritis (RA), among others [5,6]. Moreover, cell stress and ECM degradation promote maladaptive healing reactions, including inflammatory pathways of innate and adaptive immunity [7]. The loss of the biomechanical properties of cartilage induced by prior injuries or increased loading is the most significant feature in the initiation and progression of OA. Among the fibrillary components of ECM, the glycoprotein fibronectin (Fn) is an important member that acts as a bridging molecule in matrix assembly and cell–matrix interfaces. During the development of OA, tissue proteolysis and injury induce ECM degradation generating Fn fragments (Fn-fs), among other catabolic mediators, that promote inflammation and degradation by the induction of cytokine [8] and proteinase expressions [5]. This review focuses on the emerging issues related to the role of Fn-fs in the inflammatory cascade through the stimulation of urokinase-type plasminogen activator (uPA), matrix metalloproteinases (MMPs), and a disintegrin and metalloproteinase with thrombospondin motifs (ADAMTSs) proteinases in OA. The mechanisms underlying these proteinases are also discussed.

## 2. Fibronectin

**Figure 1:**
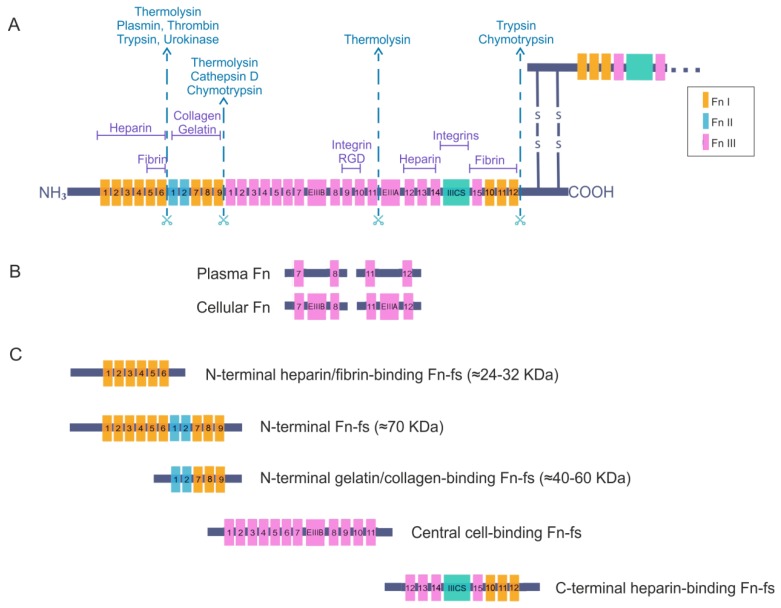
Fibronectin (Fn) and Fn fragments (Fn-fs) primary structure. (**A**) the schematic shows a representation of an Fn dimer and its interactions with different cell–surface receptors or other extracellular matrix (ECM) components such as heparin, collagen, and proteoglycans. Twelve Fn type I (FnI), two Fn type II (FnII) and fifteen constitutively expressed and two alternatively spliced Fn type III (FnIII) are indicated. The extra domains A and B (EIIIA and EIIIB) and the segment connecting two other FnIII repeats called the type III connecting segment (IIICS) or V domain are also shown. The scissors symbolize protease-sensitive regions of Fn and corresponding enzymes most commonly responsible for cleavage at these regions; (**B**) structural differences between plasma Fn and cellular Fn are shown; (**C**) the figure shows the different fragments of Fn and the structural domains that remain in each of them after breakage with proteolytic enzymes, adapted from [9,15,16,17].

Fibronectin is an adhesive glycoprotein, widely distributed in most ECM that regulates different cellular functions such as adhesion, motility, growth, differentiation, and opsonization [9,10]. It is a dimeric protein formed by two polypeptide chains with multiple repeated modular structures joined by two anti-parallel S-S bonds at the C-terminus. Each monomer of the Fn dimer has a molecular weight of 230–270 kDa, and it is composed of three different types of modules: twelve Fn type I (FnI), two Fn type II (FnII) and fifteen to seventeen Fn type III (FnIII) [11,12,13,14,15] (Figure 1A). FnI, FnII, and FnIII domains are made up of 40, 60, and 90 amino acids, respectively. Twenty different Fn proteins are observed in humans even though Fn is encoded by a single 75 kb gene [16]. This protein diversity is obtained by alternative splicing of two type FnIII exons, called extra domains A and B (EIIIA and EIIIB) and by a segment connecting two other FnIII repeats, FnIII14 and FnIII15, called the type III connecting segment (IIICS) or V domain, that can be assembled in four different ways or fully omitted. The multimodular structure and intermodular regions permit flexibility of the Fn molecule, which is involved in regulating its functions [13,15,16]. The different specific domains of Fn can interact with multiple binding partners, for example cell-surface receptors or other ECM components such as heparin, collagen, and proteoglycans. Fn is structured into four functional domains including a N-terminal domain (FnI1-9 plus FnII1-2), which binds Fn to heparin, collagen or fibrin; a central binding domain (FnII1-12) is responsible in part for Fn binding with the cells through the interaction with different integrins; and a C-terminal binding domain (FnIII12-14 plus IIICS plus FnI10-12) that also has other cell-binding sites, heparin- or fibrin-binding sites and the disulfide bridges responsible for Fn dimerization. For all of these reasons, Fn is considered to be a key molecule in the control of cellular regulatory processes and an essential scaffolding protein to preserve and maintain tissue organization [15]. 

There are two forms of Fn based on its solubility: plasma Fn, the soluble form, and cellular Fn, the water-insoluble form. The latter appears on cell surfaces and in the ECM of various tissues such as the synovial membrane and cartilage, but also in the synovial fluid. Moreover, plasma Fn has been reported to be integrated into the ECM with cellular Fn. Plasma Fn and cellular Fn have different structures and forms of assembly in three-dimensional networks (Figure 1B). Whereas plasma Fn lacks the alternatively spliced EIIIA and EIIIB regions, cellular Fn has different parts of these domains. In addition, only one subunit of plasma Fn possesses an IIICS segment. Taking these data into consideration, the number of different cellular Fn isoforms that can be generated is much higher than plasma Fn, due to the presence of alternative variants in the EIIIA, EIIIB, and IIICS segments [15,16].

Plasma Fn is synthesized by hepatocytes and directly released into circulation in a soluble, compact and inactive form. Blood plasma Fn levels increase after inflammation, major trauma, or pathologies such as atherosclerosis, ischaemic heart disease, and stroke [18,19,20,21]. Many cell types produce cellular Fn, including fibroblasts, endothelial cells, myocytes, chondrocytes, and synovial cells [15]. The different isoforms of cellular Fn are tissue-dependent, temporally regulated, and cell-type-specific. These isoforms regulate the properties of the ECM and consequently modulate different cellular processes. Under certain pathological conditions, some of the isoforms are exceptionally synthesized by cells or undergo a considerable increase in their synthesis. For instance, the isoform EIIIA is increased in synovial fluid of RA joints and correlates with the progression of joint destruction [22]. Another example is the presence of different synovial IIICS (+) isoforms, which vary their expression levels according to the degree of cartilage degeneration in OA [23]. An additional important fact to take into account is that the presence or absence of the different EIIIA, EIIIB, or IIISC regions affects the orientation and flexibility of the rest of the FnIII modules, altering the three-dimensional structure of the Fn and therefore its interactions during matrix assembly that can modulate Fn-cell signaling [15,24].

The functional form of Fn in vivo is its fibrillary state; thus, Fn molecules must be assembled into supermolecular fibers that form an interconnected network [14]. The presence of cells is necessary for Fn-matrix assembly, which occurs in several stages. First connections between Fn and cells are established through surface receptors such as integrins. Then, Fn is unfolded resulting in the exposure of Fn binding sites that allow Fn-Fn intermolecular interactions. Finally, the Fn-matrix assembly is formed. Both the expression of Fn and its assembly is regulated by a multitude of molecules in a cell-specific manner [15]. The Fn matrix assembly occurs at times of dynamic tissue remodeling, formation or repair and is essential during embryonic development [14].

### Fibronectin Fragments

Proteases play a major role during infection or inflammation and are responsible for generating different Fn-fs. Fn-fs have different functions in tissues undergoing active remodeling and can also serve as self-modulators of Fn activity [17]. Some effects of Fn-fs may be predicted by knowing where proteolysis occurs and therefore which fragments are generated. The functions of Fn-fs may differ from those of the entire Fn molecule, given that some of the domains are lost after proteolysis, secondary and tertiary structures of the molecule may be altered or active encrypted sites may appear. In addition, size reduction and altered secondary configuration may allow greater penetration into the tissues [25]. 

The Fn-fs described are classified as follows: the 70 kDa N-terminal Fn-fs (including a 24–32 kDa N-terminal heparin/fibrin-binding Fn-fs and a 40–60 kDa N-terminal gelatin/collagen-binding Fn-fs), the central cell-binding domain Fn-fs, and the C-terminal heparin binding Fn-fs (Figure 1C).

The 70 kDa N-terminal Fn-fs is a large fragment less frequent than the other two N-terminal Fn-fs, as has an interior protease-sensitive region. In fact, this can only be produced by limited exposure to cathepsin D. The size of the N-terminal heparin/fibrin-binding Fn-fs can range from 24–32 kDa. This fragment has affinity for heparin and fibrin and can form a fibrillar matrix through binding with each other at their amino-terminal end. These interactions are important in metastasic invasion or synovial invasion of cartilage. The 40–60 kDa N-terminal gelatin/collagen-binding Fn-fs, in addition to collagen or gelatin, also has affinity for C1q and tPA [17]. The central cell-binding domain Fn-fs contains an Arg-Gly-Asp (RGD) sequence, responsible for the cell-adhesive properties through the binding to different integrins. The size of this Fn-fs depends on different isoforms generated by alternatively spliced EIIIA and EIIIB regions [17,26]. For the same reason as the previous fragment, the size of the C-terminal heparin-binding Fn-fs is variable thanks to the alternative spliced IIICS region. This last fragment is responsible for cell binding through integrins and the binding to proteoglycan [17]. Little is known about the involvement of the last two fragments in different pathologies. 

Both Fn and its degradation fragments trigger cellular responses through binding to their surface receptors such as different integrins, TLRs, and CD44. In this way, they are able to activate different signaling pathways such as the MAPK pathway or transcription factors such as NF-κB or AP-1 [9,27,28,29,30,31]. In addition, they are able to establish a positive feedback with the Wnt/β-catenin pathway through integrins and MAPK signaling [32,33].

## 3. Osteoarthritis

Osteoarthritis is a chronic degenerative rheumatic disease, characterized by a progressive loss of synovial joint function as a consequence of the degradation of articular cartilage and associated with alterations in synovial membranes and subchondral bone [3,34,35]. Currently, it is considered the clinical endpoint of heterogeneous disorders with common clinical, pathological and radiological characteristics, resulting in the degeneration of one or more joints [3,36,37]. The main joints damaged are the knee, hand, spine, foot and hip [38,39]. Although it is usually an age-related disease, it is not a direct and inevitable consequence of aging. Rather, OA is also associated with other risk factors that culminate in joint dysfunction. 

OA is the most prevalent disease in developed countries and the main cause of incapacity in the elderly population. In a recent review about the socioeconomic cost of OA, it has been estimated between 0.25% and 0.50% of the gross domestic product of a country [40]. The majority of available therapies focus on relieving symptoms, but there are challenges to slowing the progression of the disease. Therefore, it is important to find new therapeutic targets aimed at the development of new drugs for the treatment of OA to mitigate the disease impact [41,42].

During the development of OA, degeneration of all joint constituents including cartilage, synovial membrane, bone, bone marrow and ligaments occurs [36,43]. The biological imbalance and the mechanical stress lead to a pathological situation with altered chondrocyte behavior releasing inflammatory mediators and ECM destruction enzymes [35,44,45,46]. All of this together with the inhibition of cartilage biosynthesis [47] increases the fragility and loss of cartilage integrity. Regarding synovial tissue, synovitis is characterized by synovial hypertrophy and hyperplasia, with increased proliferation of synovial fibroblasts (SF) and macrophage, angiogenesis and inflammatory infiltration of mononuclear cells. Although synovitis is usually localized and may even be asymptomatic [48], synovial activation causes the release of cytokines, chemokines, proteases, growth factors and other inflammatory mediators that accelerate the progression of the disease [36,49,50]. In addition, the subchondral bone also seems to play an important role in the onset and progression of OA, by releasing catabolic mediators that promote an altered metabolism in chondrocytes [39,51].

In this scenario, the degeneration of the ECM, the main component of most synovial joint tissues, contributes to the pathological process. The ECM of articular cartilage is composed of a network of type II collagen, lesser amounts of other important collagens (VI, IX, X, XI), proteoglycans and non-collagenous proteins. During the progression of OA, chondrocytes reshape the ECM of the cartilage under inflammatory conditions causing numerous changes in its mechanical function associated with increased catabolic activity [52]. This disruption of matrix equilibrium leads to progressive loss of cartilage tissue in which extracellular matrix proteases, including ADAMTSs, MMPs, and uPA, among others, play a crucial role [35,44,45,46]. Products derived from the degradation of the ECM such as collagen, Fn or its degradation fragments, induce the activation of joint cell receptors, initiating a series of signaling pathways that converge in the production of new activation and destruction mediators [49,53]. Knowledge of these proteases is essential to understand OA pathophysiology.

### 3.1. Proteases in Osteoarthritis

**Scheme 1:**
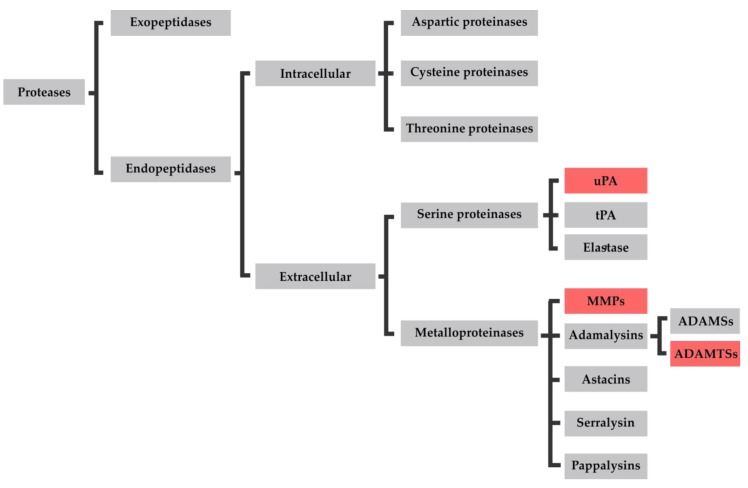
Classification of proteases. Families of proteases involved in osteoarthritis (OA) development are highlighted in red, adapted from [55,56].

Proteases are enzymes responsible for proteolytic cleavage mediated by hydrolysis of peptide bonds into the target molecule. They are involved in several biological processes, as well as in the processing and activation of precursors implicated in ECM remodeling, the immune system, development, and apoptosis. Overall, proteases can be divided into exopeptidases, when they cleave substrates at the N-terminal or C-terminal positions, and endopeptidases or proteinases, which cleave peptide bonds in the middle of the molecule. In turn, proteinases are classified according to the catalytic mechanism involved in the process of hydrolysis, where they can act intracellularly or extracellularly (Scheme 1). Among them, some extracellular proteinases, including serine proteinases and metalloproteinases, play a main role in the degradative and inflammatory processes that take place in OA development, as they are involved in cartilage destruction, bone erosion and synovial inflammation. As a consequence, they induce the release of ECM components to the OA joint, which in turn induce the expression of proteinases, therefore aggravating the pathology [54,55]. 

Proteinases released by articular cells during OA development play a key role in the degradation of the cartilage ECM leading to a loss of cartilage, a main feature in OA pathology [54,57,58]. Cartilage ECM is composed of proteoglycans, primarily aggrecan, in addition to other minor proteoglycans, such as decorin, lumican, and biglycan; adhesive glycoproteins, including the Fn; and collagens, mainly fibrillar type II collagen and other minor collagens like type IX, XI, and VI collagens [59]. Aggrecan is the main proteoglycan in cartilage ECM. It is composed of a core protein with three globular domains: G1, which interacts with hyaluronic acid and link proteins, G2, and G3, and a large extended region between G2 and G3 for glycosaminoglycan (GAGs) chain attachment, including chondroitin sulphate and keratin sulphate. These GAGs draw water into the cartilage ECM, allowing it to resist compression [54,60]. On the other hand, type II collagen is the primary collagen in cartilage ECM, which forms a fibrillar network and provides cartilage with tensile strength. Aggrecan degradation is an early event in OA progression, after which collagen degradation occurs and the process becomes irreversible [54]. Among the proteinases involved in OA, ADAMTSs, and MMPs are crucial, as they include aggrecanases and collagenases that degrade these two major structural components of cartilage ECM, the aggrecan, and the type II collagen [58]. However, other proteinases are also implicated, including other adamalysins and uPA. 

#### 3.1.1. uPA

To date, two main plasminogen activators (PAs) are known in mammals, belonging to the serine-proteinase family: the tissue-type plasminogen activator (tPA), encoded by the *PLAT* gene, and the uPA, encoded by *PLAU.* While tPA mainly acts on polymerized fibrin matrices, uPA is involved in plasminogen activation on cell surfaces, taking part in ECM remodeling. Serine proteinases seem to descend from a common ancestor gene by losses and gains of protein domains. PAs are found among all vertebrates, with an origin in jawed vertebrates. Ancestral forms of PAs have also been detected in lower vertebrates without an equivalent in humans [61]. 

**Figure 2:**
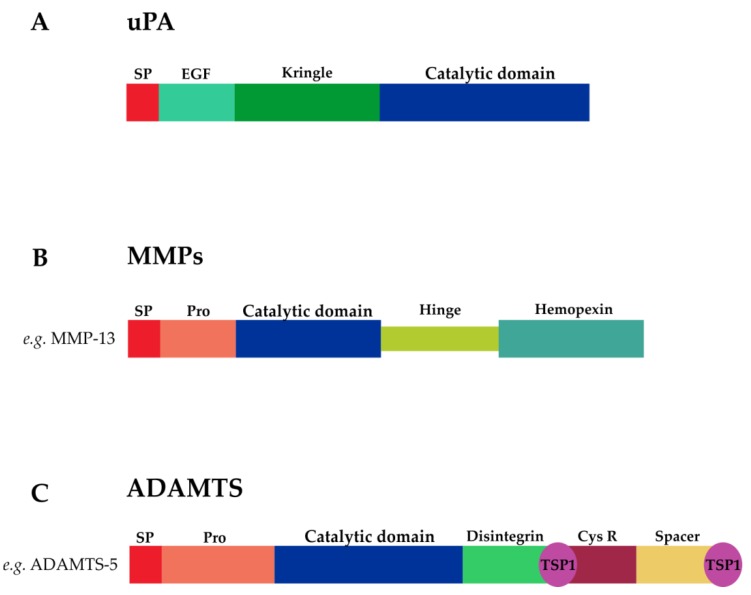
Schematic representation of proteinases involved in OA. (**A**) the serine proteinase uPA contains an N-terminal signal peptide (SP) followed by an epidermal growth factor (EGF)-like domain, a Kringle domain, and a catalytic domain. (**B**,**C**) the matrix metalloproteinase (MMP) and A disintegrin and metalloproteinase with thrombospondin motifs (ADAMTS) families have an N-terminal signal peptide, a pro-domain and a zinc-dependent catalytic domain. (**B**) In MMPs, the catalytic domain is followed by an hemopexin like-domain, (**C**) whereas, in the ADAMTS family, it is followed by a disintegrin domain and an auxiliary domain composed of a variable number of Thrombospondin type 1 (TSP-1), a cysteine-rich region (Cys R) and a spacer region, adapted from [54].

uPA is implicated in the activation of the zymogen plasminogen to plasmin. It is mainly involved in tissue remodeling and inflammation in physiologic conditions, but its deregulation is also related to several pathologies [57,62,63,64,65]. uPA is composed of a signal peptide followed by a N-terminal fragment (ATF) for receptor binding, which in turn consists of an epidermal growth factor (EGF)-like domain and a *Kringle* domain, and a C-terminal catalytic serine proteinase domain [54,65,66] (Figure 2A).

**Figure 3:**
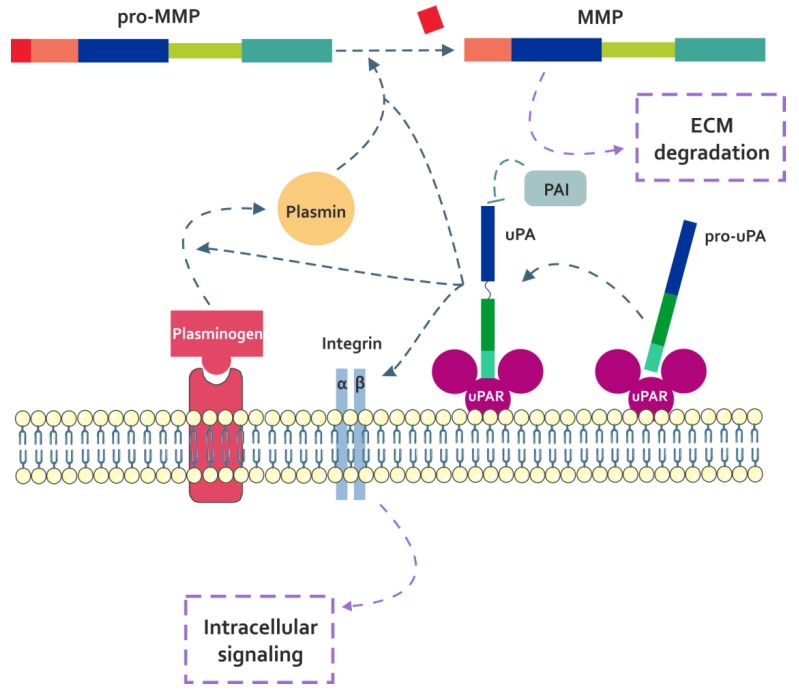
Function and regulation of a urokinase-type plasminogen activator (uPA) system. Plasmin cleaves pro-uPA generating active uPA, which, in turn, mediates the cleavage from the zymogen plasminogen to plasmin. Plasmin as well as uPA cleave pro-MMPs, generating activated MMPs, and promoting ECM degradation. In addition, uPA-urokinase-type plasminogen activator receptor (uPAR) binding interacts with integrins inducing different intracellular signaling pathways. On the other hand, plasminogen activator inhibitor (PAI) antagonizes uPA, inhibiting its activity, adapted from [56,57].

uPA is secreted as a 54 kDa single chain inactive precursor, pro-uPA, which is activated into the two-chain form after proteolytic cleavage mediated by plasmin. Its receptor, uPAR, belongs to the lymphocyte antigen 6 (Ly-6) superfamily, characterized by a Ly-6 and uPAR (LU) domain, composed of three LU domains (D1–D3) connected by short linker regions. uPAR is an important regulator of the uPA system, associated with the plasma membrane by a glycosylphosphatidylinositol anchor, which localizes pro-uPA and uPA to the cell surface. Binding of active uPA to uPAR induces the cleavage of the zymogen plasminogen to the active protease plasmin, which, in turn, cleaves and activates other proteases, including pro-MMPs in addition to pro-uPA, thereby promoting an extracellular proteolytic cascade [57,65,67]. Likewise, uPA is also able to direct activate MMPs [55]. uPA-uPAR binding activates different signaling pathways involved in several biological processes including adhesion, proliferation, differentiation, survival, chemotaxis, and phagocytosis, many of them related to innate immunity mechanisms, as well as angiogenesis, healing, and ovulation [57,64,68,69]. These pathways are also implicated in the fibrillogenesis of Fn. While uPAR is able to interact with β1, β2, β3, and β5 integrins, it seems to have higher affinity for the Fn receptors α3β1- and α5β1-integrins, which in turn act as co-receptors. In addition, uPAR interacts with other receptors, including G protein-coupled receptors (GPCRs) and the EGF-receptor. Other ligands for uPAR are the ECM protein vitronectin and the high molecular weight kininogen. On the other hand, the uPA system is negatively regulated by plasminogen activator inhibitors (PAI) or serpins. The main PAIs are PAI-1 (or serpinE1) and PAI-2 (or serpinB2). In addition, α2 antiplasmin also regulates the system through the blockage of plasmin activity [57,65] (Figure 3). 

Chondrocytes, SF, monocytes, and macrophages constitutively express uPA, but its expression can be induced by cytokines and growth factors [70,71,72,73,74,75]. Likewise, uPAR is found on the surface of joint cells, including chondrocytes, SF, and leukocytes [57,64,76]. uPA, uPAR and PAI-1 expressions are increased in inflammation and ECM remodeling processes [57,64,65,77]. uPAR is also involved in the regulation of the inflammatory response mediated by Toll-like receptor 2 (TLR2) [68]. Moreover, the uPA system is related to the development of cancer and rheumatic and inflammatory diseases, including OA and RA [63,65,78,79]. In this regard, SF from OA and RA patients express uPAR and produce PAI-1 and increased uPA levels, being involved in inflammation and joint destruction [63,65,69,78,80,81,82]. Furthermore, higher levels of uPA, uPAR, and PAI-1 have been detected in synovial fluid and a synovial tissue of OA and RA patients in comparison to normal control samples [63,83]. In addition, uPA concentration and plasmin activity were greater in OA cartilage compared to controls [73]. uPA is also capable of acting independently from uPAR, taking part in the inflammatory process in RA [69], where pro-inflammatory cytokines stimulate uPA synthesis [84].

#### 3.1.2. MMPs

MMPs and ADAMTSs belong to the metzincins superfamily, characterized as zinc-dependent enzymes responsible for ECM protein turnover, degrading type II collagen, and aggrecan [9,58]. Their action is regulated by endogen inhibitors, the tissue inhibitors of metalloproteinases (TIMP-1 to 4). While TIMP-1 and TIMP-2 are the major inhibitors of MMP action, TIMP-3 is mainly involved in the inhibition of aggrecanases [85,86]. Altered levels of MMPs, ADAMTSs and TIMPs have been described in OA patients [87,88,89].

First described in 1962 due to their collagenolytic action in tadpole metamorphosis, the MMP family is composed of 25 members, which are involved in ECM remodeling, as well as in the processing of other proteinases and their inhibitors, cell surface receptors, and growth factors. In addition, they participate in several physiological processes, including cell adhesion, proliferation, migration, apoptosis, and healing [87,90,91]. MMPs are divided into five main groups according to their substrate, structure, and cell location: collagenases, gelatinases, stromelysins, matrilysins, and membrane-type matrix metalloproteinases (MT-MMPs) [55,87,91,92,93] (Table 1). 

MMPs are found in all animal kingdoms. The MMPs identified in vertebrates are structurally similar to each other, indicating that they arose by duplication of a common ancestral gene followed by divergent evolution [94]. The phylogenetic analysis comparing human MMPs with their ortholog genes in the chordate *Ciona intestinalis*, recently recognized as *Ciona robusta* [95] and considered the closest invertebrate relatives of vertebrates, revealed the presence of six MMPs in this species: MMP-21, MMP-24, MMP-24-2 MMP-14, MMP-14-2, and only one equivalent to gelatinase MMP-9 *like* [96].

In general, MMPs are composed of a signal peptide essential for the maturation and enzyme release from the cell, a pro-domain responsible for keeping the enzyme inactive, a catalytic domain containing the Zn^2+^ atom that provides the enzymatic activity, a binding domain of variable range that is lost in some MMPs, and a hemopexin domain that is also lost in some MMPs, lending substrate specificity in addition to the interaction with some inhibitors and cell receptors [55,87,92,93] (Figure 2B). Due to their proteolytic activity, the MMPs are strongly regulated and require the action of other enzymes for removal of the pro-domain and activation, including MT-MMPs, cathepsin B, plasmin, uPA, and tPA. This activation is usually extracellular, but can also occur at the cell surface, such as for MMP-13 and MMP-2, or intracellularly in the Golgi complex, as in the case of MMP-11 and MT-MMPs [97]. 

An exacerbated activation of MMPs can lead to the development of several pathologies, including cancer, cardiovascular diseases, and inflammatory and/or autoimmune diseases, such as OA and RA. In this regard, increased levels of MMPs have been detected in the synovial tissue and cartilage of OA and RA patients [59,88,89,93,98,99,100]. 

##### Collagenases

Among the MMPs involved in OA, the collagenases play a key role in degrading collagen from the cartilage ECM. This group is composed of MMP-1 (collagenase-1), MMP-8 (collagenase-2), MMP-13 (collagenase-3), and MMP-18, which can be released by chondrocytes, as well as by SF and synovial macrophages in the joint microenvironment. Both MMP-1 and MMP-13 are central to OA pathology. While MMP-1 is predominantly produced by SF and is involved in early stages of the disease contributing to the destruction of the cartilage surface, MMP-13, mainly released by chondrocytes, is essential in successive phases of ECM remodeling [59,101]. 

The role of MMP-13 is crucial for OA development and progression, as it is the main factor involved in the degradation of type II collagen, along with other ECM components, including aggrecan [58,59,87,92,102,103]. In addition to articular cartilage degradation, MMP-13 also causes its erosion and ulceration. Furthermore, it is involved in synovial inflammation, inducing synovial hyperplasia with mononuclear cell infiltration in the joint [103]. Increased levels of MMP-13 have been detected in cartilage from OA patients, where it is associated with a greater destruction [104,105,106], as well as in SF from RA patients, correlating with uPA levels [107]. MMP-13 is also present in SF and synovial fluid from OA and RA patients [108,109,110,111], where cytokines such as IL-1β and other inflammatory mediators are involved in the induction of its expression [80,92].

##### Gelatinases

Increased levels of other MMPs have also been described in OA and other rheumatic disease, including MMP-2 and MMP-9 [59]. MMP-2 and MMP-9 belong to the group of gelatinases, which have a broad range of substrate specificity. The gelatinases not only degrade gelatins, but also other ECM components, including Fn and different types of collagen. In this regard, MMP-2 is able to degrade type I, II, and III collagens, but with a lower activity than collagenases. Gelatinases are produced by mesenchymal cells, macrophages and mononuclear cells from peripheral blood, but can also be released from activated and tumoral cells. As a special feature of their structure, gelatinases have three Fn type II repeat domains in their catalytic domain, which facilitate substrate binding and degradation [87,99]. 

Among the gelatinases, MMP-9 or gelatinase B is involved in physiological and pathological processes, being implicated in tumoral cell survival, as well as in cardiovascular, degenerative, autoimmune, and inflammatory diseases, where MMP-9 activates pro-inflammatory mediators reciprocally. This gelatinase is characterized by a flexible O-glycosylated domain between the catalytic and hemopexin domains, which is essential for its function [92,112]. MMP-9 levels in synovial fluid from OA and RA patients are related to MMP-13 and uPA, taking part in the progression of the disease [107].

##### Other MMPs

Stromelysins show a wide range of substrate specificity, including proteoglycans, Fn, elastin and laminin, among other ECM components [92]. Among them, MMP-3 is one of the MMPs most expressed in OA cartilage, which decreases in late phases of the disease [113]. Increased levels of MMP-3 have been described in arthritic diseases [59], suggesting an important role of this MMP in the release of proteoglycans from cartilage [114]. 

Regarding matrilysins, MMP-7 is able to degrade different ECM components, also including proteoglycans [115]. Higher levels of MMP-7 have been observed in OA synoviocytes compared to controls in a rat model of knee OA [116]. In addition, overexpression of this MMP has been detected in human OA cartilage [115]. 

Finally, the MT-MMPs subfamily of MMPs is characterized by a transmembrane domain at the C-terminus. The MT-MMPs act at the cell surface and are able to activate other MMPs, suggesting a role in ECM degradation as well. Among them, MT1-MMP also has collagenolytic activity, and is expressed in human articular cartilage, synovial membrane, and osteoclast-like cells in bone resorption. MT3-MMP is highly expressed in OA and RA synovium as well. On the other hand, MT2- and MT4-MMP do not seem to be involved in joint destruction [92,117]. 

#### 3.1.3. ADAMTSs

The ADAMTS superfamily includes secreted metalloproteases, ADAMTS proteases, as well as ADAMTS-like proteins (ADAMTSLs), structurally-linked secreted glycoproteins that lack catalytic activity. Members of both families contribute to varied morphogenetic processes during embryonic development, and connective tissue maintenance. Research on ADAMTSLs proteins is less advanced, although it seems that they are functionally related to ADAMTSs, such as in the turnover and assembly of fibrillin microfibrils [118,119].

The ADAMTS family includes zinc-dependent endopeptidases that play an essential role in developmental processes and in the maintenance of homeostasis by remodeling ECM [120]. Alterations in ADAMTS expression are also related to the development of certain inflammatory, arthritic, vascular and neurodegenerative pathologies, as well as cancer [121]. The first ADAMTS was discovered in the late 1990s [122].

There are 19 ADAMTS genes in humans, designated ADAMTS1 through ADAMTS20, where ADAMTS5 and ADAMTS11 represent the same gene. The biology of a protease is based on the substrates on which they perform their proteolytic activity. Therefore, the remaining 18 members of proteases are sub-classified according to their specific substrate in five subgroups [123,124,125] (Table 2). Phylogenetically, in invertebrates, only one ADAMTS, called Gon-1, with a common ancestor within the same clade as ADAMTS9 and ADAMTS20 in mammals, has been described in nematodes [126]. The phylogenetic analysis comparing human ADAMTSs with their ortholog genes in the chordate *C. robusta*, revealed different evolutionary branches of ADAMTS proteases that originated by duplication of ancestral genes represented by six *C. robusta* orthologs [125]. It has also been proposed that, after duplication, vertebrate paralogues have mainly developed through sub-functionalization, rather than neo-functionalization. Moreover, data from vertebrates support the ADAMTS gene family evolution through duplication processes across metazoans, complemented by a burst of amplification through vertebrate whole genome duplication events [127]. Therefore, there are robust data to speculate that the core genes of protease activity have been conserved in the phylogeny and predict the functional sub-classification of human ADAMTSs.

ADAMTSs are composed of a proteinase domain and a C-terminal auxiliary domain, which provides the substrate specificity, allows the interaction with the ECM, regulates its activity and ultimately determines its location. The proteinase domain is composed of a signal peptide, a pro-domain, a catalytic domain and a disintegrating domain. The auxiliary domain is formed by a variable number of repeated motifs of thrombospondin type 1 (TSP-1), a cysteine-rich region and a spacer region. The carboxyl terminal may vary depending on the enzyme, and may contain other TSP-1 domains and motifs [123,128] (Figure 2C).

Multiple isoforms of ADAMTSs generated by alternative splicing processes and post-translational modifications of glycosylation and proteolysis, involved in their secretion, location, activation and catalytic function, have been reported [123,129,130]. Like MMPs, ADAMTSs are synthesized inactively and released as zymogens. These zymogens undergo N-terminal processing, which removes the signal peptide in addition to the pro-domain, and allows accurate protein processing and secretion. The pro-domain usually contains at least one binding site for furin or furin-like proprotein convertases, which are responsible for intracellular or extracellular activation of the enzyme by its elimination. Nevertheless, certain aggrecanases do not need the removal of the pro-domain to be active. Once secreted, ADAMTSs may also undergo processing at the C-terminal end, where the spacer region is involved [123,131,132,133].

##### Aggrecanases and Proteoglycanases

The aggrecanases and proteoglycanases subgroup includes a series of ADAMTSs that are able to break chondroitin sulphate proteoglycans that bind to hyaluronic acid [134]. The aggrecan is one of the main components of the cartilage ECM, which also protects collagen from degradation. The loss of ECM aggrecans originating in the early stages of OA is one of the key events in the development of the disease [85,123]. In fact, it can be observed as degradation products in synovial fluid from patients with OA and RA [135,136]. In this process, aggrecanases take on an essential role. Although there are also MMPs and other proteases capable of degrading aggrecans, in contrast to aggrecanases, they cannot cleave it at the highly-conserved sequence of the protein nucleus involved in the pathogenesis of OA, NITEGE373-374ARGSV of the intraglobular domain, between Glu373 and Ala374 [85,123]. This proteolysis results in the generation of the ARGSV and NITEGE neoepitopes, which are elevated in cartilage from OA and AR patients [137,138]. Moreover, this subgroup of ADAMTSs can also cleave versican, brevican, and neurocan.

ADAMTS1 breaks aggrecan and versican and is expressed in cartilage and synovium. Data about its expression in OA are controversial; some authors reported an increased expression in cartilage [139] whereas recent results using qRT-PCR showed that ADAMTS1 is downregulated in joint synovial tissues [140].

Regarding ADAMTS4 and ADAMTS5, the first critical step in OA development is the loss of cartilage aggrecans mediated by these ADAMTSs. Although ADAMTS5 is the main mouse aggrecanase, there is disagreement about which of the two aggrecanases is the most important in humans. Both are involved in the pathogenesis of OA, where they play an important role through the degradation of aggrecan, mainly in the initial and final stages [123,141]. Some studies consider ADAMTS4 the main factor implicated in OA because its levels are increased in the cartilage of these patients, correlating with a greater degradation of this tissue. However, other authors reported ADAMTS4 and -5 expressions in cartilage and SF, where ADAMTS5 was more expressed in SF from healthy donors (HD) than in OA, while ADAMTS4 gene expression was similar in both [33,50]. In addition, in vitro studies have shown that ADAMT5 activity is higher than that of ADAMTS4 [142]. It has been described that the expression of ADAMTS4 can be induced with different pro-inflammatory mediators, such as IL-1β or TNFα, while some authors point out that ADAMTS5 is constitutive [143,144]. Nevertheless, different studies indicated that both ADAMTSs can be induced [145,146]. 

Studies on ADAMTS8, ADAMTS9, ADAMTS15, and ADAMTS20 are less common. ADAMTS8 has aggrecanase activity. It is expressed in healthy cartilage and an increased expression in human OA synovium has been described. ADAMTS9 and -20 cleave aggrecan and versican, respectively. They are both expressed in healthy cartilage, being upregulated in OA. Regarding ADAMTS15, it degrades aggrecan and versican, and its expression in OA cartilage is controversial [147]. 

##### COMP Proteinases

ADAMTS7 and ADAMTS12 are the only ADAMTSs that have a mucin domain, to which the condroitin sulphate chains are bound, giving them proteoglycan characteristics [148]. They also contain a protease and lacunin (PLAC) motif at its C-terminal end, with six conserved cysteine residues. Its processing depends on furins and ends in the extracellular space. The importance of these ADAMTSs lies in their ability to degrade COMP [149], a ECM protein that contributes to the maintenance of cartilage stability through its interaction with other components, such as aggrecan, type II collagen or Fn [149,150,151]. Fragments derived from their degradation in cartilage, synovial fluid and serum have been described in patients with OA and RA [152]. The TSP-1 motifs of the C-terminal end of the ADAMTS7 and -12 allow them to interact with COMP [149,153]. In turn, COMP is associated with the precursor of granulin-epithelin (GEP), which inhibits the activity of ADAMTS7 and -12. Elevated levels of GEP in the synovial tissue of patients with OA and RA have also been described [149,154]. Likewise, alpha-2-macroglobulin (α2M) is another substrate of ADAMTS7 and -12, also involved in their regulation. On the other hand, ADAMTS12 is also capable of degrading the aggrecan of the ECM, but with a much smaller capacity than the ADAMTS4 and -5 aggrecanases [155]. ADAMTS7 and -12 are involved in the pathogenesis of different diseases, including rheumatic and vascular diseases and cancer [123,149]. Both enzymes are expressed in the joint, including cartilage, bone and synovial tissue. ADAMTS7 and -12 also participate in the inhibition of chondrogenesis, acting as targets of the parathyroid hormone-related protein [149,153]. High levels of these ADAMTSs have been described in patients with OA and RA [88,156,157], which can be induced by pro-inflammatory stimuli, although in the case of ADAMTS12, this depends on the cell type involved [158]. Moreover, COMP degradation in OA cartilage explants is inhibited by neutralizing antibodies against both ADAMTSs [155].

ADAMTS7 and ADAMTS12 have been detected in joint cartilage with a higher expression in OA than in HD. Moreover, we have described the expression and release of both ADAMTSs in SF, which represent a source of metalloproteinases for the perpetuation of cartilage damage [33,50].

##### Procollagen N-Propeptidases

Synthesis of collagen fibers is a complex process. Fibrillar collagens are secreted as pro-molecules composed of a large central triple helical domain extended by propeptides at both extremities. These amino- and carboxy-propeptides are then cleaved by procollagen N-proteinases and C-proteinases, respectively, reducing the solubility of the collagen molecules, which induces the spontaneous assemblage into lengthened collagen fibrils.

ADAMTS2, ADAMTS3, and ADAMTS14 are responsible for the cleavage of the N-terminal propeptide of the fibrillary collagens. ADAMTS2 was first described in dermatosparaxis, a cattle disease characterized by extreme skin fragility [159]. Subsequently, dermatosparaxis Ehlers–Danlos syndrome (dEDS) was identified as the equivalent in humans, characterized by the presence of different mutations in the ADAMTS2 gene [160].

An elevated expression of ADAMTS2 has been identified in all type I collagen-rich tissues from fetal calf such as skin, bones, tendons and aorta, supporting its significance in type I collagen assembly [161]. Increased expression of ADAMTS2 has been described in OA cartilage [147]. Moreover, this ADAMTS has been demonstrated to exhibit effective anti-angiogenic activity [162].

ADAMTS3 is mainly expressed in cartilage, where it colocalizes with type II procollagen, and in the nervous system [163,164]. It is more effective than ADAMTS2 in processing type II pro-collagen [163]. An augmented expression of this protease along with ADAMTS14 has also been described in OA [165,166]. In this regard, two polymorphisms have been identified in the ADAMTS14 gene associated with an increased risk of knee OA in woman [167,168]. However, the role played by these proteases in rheumatic pathologies is still quite unknown and more studies are needed.

##### von-Willebrand Factor Proteinase

ADAMTS13 is a von-Willebrand factor-cleaving proteinase [123]. Regarding OA, no data have been described about this ADAMTS; only its upregulation in human OA synovium has been reported [89].

##### Other ADAMTSs

Finally, another group of ADAMTSs with an unknown substrate have been called “orphan” proteases. ADAMTS6, ADAMTS10, and ADAMTS18 are expressed in OA cartilage. ADAMTS6 and -10 have been involved in the regulation of cell–cell junctions and focal adhesion [169]. ADAMTS6 transforms procollagen to collagen [170]. An inguinal hernia genetic susceptibility locus near the ADAMTS6 gene has been reported, indicating that collagen deregulation could be involved in the progress of inguinal hernias [171]. Mutations in the ADAMTS10 gene have been reported in Weill–Marchesani syndrome (WMS), a disorder characterized by abnormalities in the eye and skeletal development [172]. Finally, regarding ADAMTS16 and -17, although no data correlating these ADAMTs to rheumatic pathologies have been found, they are involved in bone development and collagen processing. Thus, three mutations in the ADAMTS17 gene have been identified supporting a role in the development of a connective-tissue disorder that is similar to WMS [172]. Genetic variants of ADAMTS16 and -19 associated with inherited hypertension and premature ovarian failure, respectively, have been reported [121].

Only by understanding the full spectrum of proteinases expressed in the joint and their biological functions in this location will it be possible to design strategies to selectively target pathological tissue destruction.

## 4. Fibronectin and Fibronectin Fragments in Osteoarthritis

High levels of Fn have been described in the superficial layer of OA articular cartilage. Moreover, elevated levels of Fn accumulate in the inflamed synovial tissue and in the articular cartilage of RA joints. Enhanced levels of Fn are also observed in the synovial fluid, being 55% higher than levels in normal plasma [9,173,174,175]. In the course of these rheumatic diseases, in addition to the enhanced amounts of Fn in the joints, an exacerbated activation of Fn-degrading proteases has been described. In fact, increased levels of Fn-fs have been reported in the synovial fluid of OA and RA patients [9,17,176,177]. These fragments are included within the term matrikines, peptides originated from the fragmentation of ECM proteins that play an important role in both health and disease [178]. The activity of proteases is crucial during infection and inflammation, being responsible for the generation of different Fn-fs. 

Fn-fs have properties not present in native Fn, and are the main candidates for the maintenance of cartilage destruction and synovial tissue inflammation in OA. Thus, 29-, 45-, 120-, and 200 kDa Fn-fs derived from Fn have been found in OA cartilage and synovial fluid, where they stimulate the production of various inflammatory cytokines, such as TNFα and IL-1β [114]. In addition, increased release of 29 kDa N-terminal heparin-binding, 50 kDa N-terminal gelatin-binding, and 110-140 kDa C-terminal heparin-binding Fn-fs have been detected in bovine injured cartilage explants compared to controls [179]. More recently, it has been reported that 29 kDa N-terminal heparin-binding Fn-fs inhibited autophagy through modifying localization of HMGB1 in human articular chondrocytes [180].

Along with OA, another musculoskeletal pathology with a huge worldwide prevalence is low back pain, which is generated by the degenerative disc disease, among other causes [181]. The degenerative progression in intervertebral discs is generally identified as intervertebral disc degeneration (IVD) [182]. IVD and OA share characteristics and properties such as cell physiology and ECM of the nucleus pulposus and articular cartilage. It has been suggested that, in acute stages, IVD is accompanied by loss of joint space, subchondral sclerosis, and osteophytes, comparable to OA in the articular joint. As occurs in OA, in IVD local inflammation is the result of mechanical overloading or low grade systemic inflammation, being characterized by increased levels of inflammatory cytokines. Moreover, this inflammatory cascade produces the degradation of ECM with the involvement of different proteinases including MMP-1–3, -7–10, and -12–14, as well as ADAMTS4 and -5. As a result, in both diseases, a chronic inflammation loop is established that degenerates both intervertebral disc and articular joint [183]. An additional element of similarity is the role that Fn plays in both pathologies. In this sense, an increased expression of Fn and Fn-fs has been reported in human spontaneously degenerative discs [184]. Moreover, the high levels of Fn-fs have been related with disc degeneration mediated by upregulation of MMP-3 and MMP-13 [29]. Ruel et al. have described that adult IVD tissues contains many Fn-fs that were absent from the infant disc tissue (6). The highest amount of FN-fs was present in the moderately degenerative discs, as well as in the initial phases of disc degeneration, before observable changes to the disc tissue arise, thus suggesting that Fn-fs play a significant role in the initiation and evolution of disc degeneration [185].

Baker et al. proposed a mathematical model showing that positive and negative feedback monitoring the degree of cytokine production can regulate either the pre-disposition to OA or the initiation of OA. Moreover, they proposed that manipulation of cytokines, proteinases and Fn-fs levels could be used to treat OA and other related pathologies, suggesting that multiple treatment targets may be essential to break or slow disease progression [186]. 

In the next section, we describe the multi-target effect of different Fn-fs in the modulation of ECM-degrading enzymes in OA.

### 4.1. Effect of Fibronectin Fragments in the Profile of OA Matrix-Remodeling Proteinases

**Figure 4:**
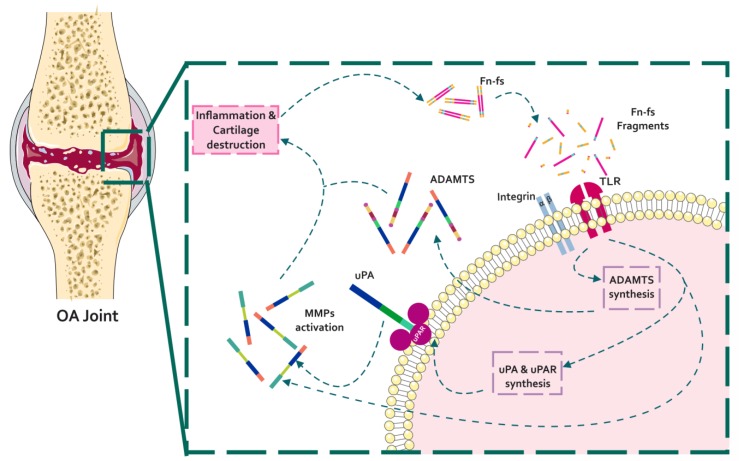
Schematic representation of the interaction of Fn with proteinases in the context of OA pathology. In a situation of inflammation and cartilage damage, Fn cleavage from the cartilage ECM promotes the release of Fn-fs to the joint microenvironment. Binding of Fn-fs to their receptors (integrins and TLRs) activates different signaling pathways increasing the expression of proteinases (uPA, MMPs, and ADAMTSs). In turn, active proteinases degrade the cartilage ECM inducing a feed-back loop of inflammation and cartilage degradation.

During OA, ECM degrading enzymes induce the release of damage-associated molecular patterns (DAMPs) with catabolic properties, including Fn-fs. These Fn-fs act as pro-inflammatory mediators in a positive feedback loop inducing the expression of pro-inflammatory cytokines, nitric oxide (NO), and other inflammatory mediators, as well as proteinases, degrading ECM components, and therefore promoting inflammation and cartilage destruction in OA [9,176,177]. In this regard, different authors have described the induction of proteinases mediated by Fn-fs in chondrocytes and SF, including uPA, MMPs, and ADAMTSs [33,80,114,174,187] (Figure 4). 

#### 4.1.1. Fn-fs Modulate the uPA System and Induce MMP Expression

Deregulation of the uPA system is related to the development of several pathologies including OA [39,54,55,92]. During this disease, Fn-fs are released to the joint microenvironment as a consequence of ECM degradation. In this respect, it has been described that 45 kDa N-terminal gelatin-binding Fn-fs increase uPA expression and activity and uPAR production in OA-SF [80]. Furthermore, 29 kDa N-terminal heparin-binding Fn-fs also increases uPA levels in bovine articular cartilage [114]. Moreover, 40- to 60 kDa N-terminal gelatin-binding Fn-fs are associated with the binding of immunoglobulins and complement proteins to inflammation sites, as well as with the activity of tPA and uPA [17,80]. In addition, these fragments have been related to proteoglycanase activity, associated with MMPs or ADAMTSs [17,33].

As mentioned above, binding of active uPA to uPAR induces the activation of plasminogen to plasmin, which in turn activates MMPs. In this regard, in addition to the uPA system, 45 kDa N-terminal gelatin-binding Fn-fs also increase the production of MMP-9 and MMP-13 in OA-SF culture supernatants [80]. Moreover, these fragments induce MMP-3 and MMP-13 synthesis in porcine cartilage [188]. Elevated release of MMPs and proteoglycans, as well as increased proteoglycan degradation and induction of NO and catabolic cytokines, has been observed in bovine articular cartilage [114]. In addition, 50% removal of articular cartilage proteoglycans mediated by Fn-fs has been described in rabbit knee joints [189]. This proteoglycan depletion is mainly associated with the activity of MMP-3, increased by 29 kDa N-terminal heparin-binding, 50 kDa N-terminal gelatin-binding, and 140 kDa central cell-binding Fn-fs in bovine cartilage. MMP-3 in turn degrades Fn, generating new Fn-fs in a positive feedback loop [114,179,190]. Furthermore, 29 kDa N-terminal heparin-binding Fn-fs also enhance gelatinase expression in bovine cartilage [114,174]. These Fn-fs have been involved in the stimulation of the expression of catabolic factors through the TLR2-dependent signaling pathway. Thus, the exposure to 29 kDa N-terminal heparin-binding Fn-fs from synovial fluid of OA patients, increased MMP-1, MMP-3, and MMP-13 expression in primary chondrocytes, where TLR2 knockdown significantly blocked the synovial fluid-induced MMP stimulation [28]. Moreover, involvement of α5 integrins has been shown in cartilage proteoglycan degradation. In this respect, α5 integrins seem to be implicated in the N-terminal heparin- and gelatin-binding, central cell-binding, and C-terminal heparin-binding Fn-fs induction of proteoglycan loss, promoting cartilage destruction in bovine cartilage [25,174,191]. 

Central cell-binding Fn-fs are able to stimulate MMP-1 and MMP-3 expression in rabbit SF [187], as well as MMP-3 in rabbit chondrocytes [192]. Moreover, 120 kDa central cell-binding Fn-fs increase MMP-13 in human chondrocytes, with the involvement of α5β1 integrins [193,194]. These 120 kDa Fn-fs also induce collagenase expression in rabbit SF [195]. In addition, 29 kDa N-terminal heparin-binding and 140 kDa central cell-binding Fn-fs, increase the tissue inhibitor of metalloproteinases TIMP-1 in bovine articular cartilage [114]. 

Finally, C-terminal heparin-binding Fn-fs induce MMP-3 and MMP-13 production in bovine cartilage [196], MMP-1, MMP-2, MMP-9, and MMP-13 in human cartilage [197], as well as MMP-1, MMP-3, and MMP-13 in RA-SF [198]. Moreover, these Fn-fs also increase type II collagen cleavage in human and bovine cartilages, mainly mediated by MMP-13 [196,197]. 

While Fn is less active than its degradation products, it is also able to induce the expression of metalloproteinases in some contexts. In this regard, Fn increases MMP-9 production in human and murine macrophages [199]. Furthermore, Fn induces MMP-2 expression in human prostate cancer cells [200]. MMPs in turn cleave Fn, generating Fn-fs. Therefore, MMP-1, MMP-3, MMP-13, and MMP-14 degrade Fn generating 70 kDa Fn-fs, where MMP-13 and -14 were the most effective. Moreover, MMP-13 and -14, are also able to generate 52-, 40-, 32-, and 29 kDa Fn-fs in articular cartilage from bovine knee joints [201]. In addition, MMP-8, -9 and -12 also cleave Fn in human articular cartilage, where MMP-12 seems to be the main metalloproteinase [202].

#### 4.1.2. Fn-fs Modifies ADAMTS Expression and Signaling

The 45 kDa N-terminal gelatin-binding Fn-fs has been shown to increase aggrecanase levels in SF. Specifically, these Fn-fs induce ADAMTS4 expression and protein production in HD- and OA-SF, ADAMTS5 expression and protein production in OA-SF, as well as aggrecanase activity in OA-SF, and GAGs release in HD- and OA-SF [33]. In addition, these 45 kDa Fn-fs induce aggrecan degradation and the generation of aggrecanase-derived neoepitopes in porcine cartilage [188]. Induction of ADAMTS5 mediated by 29 kDa N-terminal heparin-binding Fn-fs has also been described in human and bovine chondrocytes [203,204]. Furthermore, these ADAMTSs, primarily ADAMTS4, are also involved in Fn cleavage in human articular cartilage [202,205]. On the other hand, the 40 kDa C-terminal heparin-binding Fn-fs is able to inhibit the aggrecanase activity of ADAMTS4 in human chondrocytes, suggesting a positive effect of this Fn-fs by avoiding cartilage degradation [206].

Regarding COMP-degrading ADAMTSs, 45 kDa N-terminal gelatin-binding Fn-fs also increase ADAMTS7 expression and protein production in HD- and OA-SF, ADAMTS12 expression, and protein production in OA-SF, and COMP degradation in HD- and OA-SF [33]. On the other hand, concerning ECM assembly, COMP interacts with Fn, with a predominant binding site at the N-terminal domain of Fn [150].

In relation to other ADAMTSs, ADAMTS16 inhibits Fn fibrillogenesis and cleaves Fn, releasing a 30 kDa N-terminal heparin-binding Fn-fs, which in turn, upregulates MMP-3, which is also able to cleave Fn, generating new Fn-fs and inducing a positive degradative feedback loop in an epithelial cell line [207]. Additionally, ADAMTS9 is involved in Fn turnover and fibrillogenesis in ECM remodeling during mouse embryogenesis [208].

Finally, regarding signaling pathways implicated in ADAMTS expression, 45 kDa N-terminal gelatin-binding Fn-fs induce Runx2 activation in HD- and OA-SF and Wnt/β-catenin signaling in OA-SF [33,50]. Furthermore, Fn is a target of Wnt signaling in mouse embryonic lung morphogenesis [209] and, in turn, the canonical Wnt pathway is involved in Fn and metalloproteinase expression in RA-SF [210], suggesting a feedback loop between Fn and β-catenin [211].

## 5. Conclusions

- Fibronectin is a component of the ECM essential to its assembly, which also regulates some cellular functions. However, cleavage of fibronectin in pathological conditions releases fibronectin fragments with pro-inflammatory and degradative properties.
- Fibronectin fragments mainly released from cartilage ECM during osteoarthritis induce the expression of different proteinases, such as uPA, MMPs, and ADAMTSs. These proteinases degrade several ECM components, including the type II collagen and the aggrecan.
- Overexpressed proteinases in the osteoarthritis joint microenvironment cleave fibronectin, therefore releasing new fibronectin fragments and promoting a feed-back loop of degradation and inflammation in the pathology.
- The study of fibronectin and proteinases in pathological conditions is important for the design of new strategies using them as potential therapeutic targets for the treatment of osteoarthritis, to control not only the symptoms but also the progression of the disease.

## Figures and Tables

**Table 1:** Classification of human matrix metalloproteinases (MMPs).

Family	Enzyme	Main Substrates
**Collagenases**	**MMP-1 (collagenase-1)**	Collagen I, II, III, VII, VIII, X, and XI, gelatin, entactin, tenascin, aggrecan, fibronectin, vitronectin, myelin basic protein, ovostatin, casein, MMP-2, MMP-9, proMMPs
	**MMP-8 (collagenase-2)**	Collagen I, II, and III, fibronectin, prostaglandins, aggrecan, ovostatin
	**MMP-13 (collagenase-3)**	Collagen I, II, III, IV, IX, X and XIV, gelatin, tenascin, plasminogen, osteonectin, fibronectin, aggrecan, casein
	**MMP-18**	Collagen, gelatin
**Gelatinases**	**MMP-2 (gelatinase A)**	Collagen I, III, IV, V, VII and X, gelatin, fibronectin, laminin, aggrecan, elastin, vitronectin, tenascin, myelin basic protein
	**MMP-9 (gelatinase B)**	Collagen IV, V, XI, elastin, aggrecan, decorin, laminin, entactin, myelin basic protein, casein
**Stromelysins**	**MMP-3 (stromelysin-1)**	Collagen III, IV, V, IX, X and XI, gelatin, aggrecan, elastin, fibronectin, vitronectin, laminin, entactin, tenascin, decorin, myelin basic protein, ovostatin, casein, osteonectin, proMMPs
	**MMP-10 (stromelysin-2)**	Collagen III, IV and V, gelatin, elastin, fibronectin, aggrecan, casein
	**MMP-11 (stromelysin-3)**	Gelatin, fibronectin, collagen IV, laminin, elastin, casein, prostaglandins
	**MMP-27**	Gelatin
**Matrilysins**	**MMP-7**	Collagen I and IV, gelatin, elastin fibronectin, vitronectin, laminin, entactin, tenascin, aggrecan, myelin, proMMP-1, proMMP-2, proMMP-9 transferrin, casein
	**MMP-26**	Collagen IV, gelatin, fibronectin, fibrinogen, pro-MMP9
**MT-MMPs**	**MMP-14 (MT1-MMP)**	Collagen I, II and III, gelatin, fibronectin, tenascin, vitronectin, laminin, entactin, aggrecan, vibronectin, pro-MMP2
	**MMP-15 (MT2-MMP)**	Fibronectin, tenascin, entactin, laminin, aggrecan, gelatin, vibronectin, pro-MMP2
	**MMP-16 (MT3-MMP)**	Collagen III, gelatin, fibronectin, casein, laminin, pro MMP-2
	**MMP-17 (MT4-MMP)**	Gelatin, fibrinogen, pro MMP-2
	**MMP-24 (MT5-MMP)**	Fibronectin, gelatin, proteoglycans, pro-MMP2
	**MMP-25 (MT6-MMP)**	Collagen IV, gelatin, fibronectin, proteoglycans, pro-MMP2
**Other MMPs**	**MMP-12**	Collagen I, V and IV, gelatin, elastin, fibronectin, vitronectin, laminin, entactin, osteonectin, aggrecan, myelin, vitronectin, fibrinogen
	**MMP-19**	Collagen I and IV, gelatin, lamin, entactin, fibronectin, aggrecan
	**MMP-20**	Amelogenin, aggrecan
	**MMP-21**	Not defined
	**MMP-23**	Gelatin
	**MMP-28**	Caseine

MMPs are classified in families according to their main substrates, adapted from [56,91].

**Table 2:** Classification of human a disintegrin and metalloproteinase with thrombospondin motifs (ADAMTSs).

Family	Enzyme	Main Substrates
**Agrecanases and proteoglycanases**	**ADAMTS1** **ADAMTS4** **ADAMTS5** **ADAMTS8** **ADAMTS9** **ADAMTS15** **ADAMTS20**	Proteoglycans: aggrecan, versican, brevican, neurocan, fibromodulin, decorin, carboxylmethylated transferrin
**COMP proteinases**	**ADAMTS7** **ADAMTS12**	COMP
**Procollagen** **N-peptidases**	**ADAMTS2** **ADAMTS3** **ADAMTS14**	Procollagen
**von-Willebrand factor proteinases**	**ADAMTS13**	von-Willebrand coagulation factor
**Other ADAMTS**	**ADAMTS6** **ADAMTS10** **ADAMTS16** **ADAMTS17** **ADAMTS18** **ADAMTS19**	Not defined

ADAMTSs are classified in families according to their main substrates, adapted from [56].

## Abbreviations

ADAMTSLs	ADAMTS-like proteins
ADAMTSs	A disintegrin and metalloproteinase with thrombospondin motifs
DAMPs	Damage-associated molecular patterns
dEDS	Dermatosparaxis Ehlers-Danlos syndrome
ECM	Extracellular matrix
EGF	Epidermal growth factor
Fn	Fibronectin
Fn-fs	Fibronectin fragments
GAGs	Glycosaminoglycans
GEP	Granulin-epithelin
GPCRs	G protein-coupled receptors
HD	Healthy donors
IVD	Intervertebral disc degeneration
LU	Ly-6 and uPAR domain
Ly-6	Lymphocyte antigen 6
MMPs	Matrix metalloproteinases
MT-MMPs	Membrane-type matrix metalloproteinases
NO	Nitric oxide
OA	Osteoarthritis
PAI	Plasminogen activator inhibitor
PAs	Plasminogen activators
PLAC	Protease and lacunin
RA	Rheumatoid arthritis
SF	Synovial fibroblasts
TIMP	Tissue inhibitors of metalloproteinases
TLR2	Toll-like receptor 2
tPA	Tissue-type plasminogen activator
TSP-1	Thrombospondin type 1
uPA	Urokinase-type plasminogen activator
uPAR	Urokinase-type plasminogen activator receptor
WMS	Weill–Marchesani syndrome
α2M	Alpha-2-macroglobulin
